# The impact of prenatal counselling on mothers of surviving children with hypoplastic left heart syndrome: A qualitative interview study

**DOI:** 10.1111/hex.13103

**Published:** 2020-07-15

**Authors:** Sophie Bertaud, David F. A. Lloyd, Gurleen Sharland, Reza Razavi, Myra Bluebond‐Langner

**Affiliations:** ^1^ Department of Imaging Sciences and Biomedical Engineering King's College London Guy's and St Thomas' Hospital London UK; ^2^ Louis Dundas Centre for Children's Palliative Care UCL Great Ormond Street Institute of Child Health London UK; ^3^ Department of Congenital Heart Disease Evelina London Children's Hospital Guy's and St Thomas' NHS Foundation Trust London UK

**Keywords:** counselling, decision making, hypoplastic left heart syndrome, prenatal diagnosis

## Abstract

**Objective:**

To explore the role of antenatal counselling in how parents make treatment decisions following an antenatal diagnosis of Hypoplastic Left Heart Syndrome (HLHS).

**Background:**

Antenatal counselling is a critical part of patient management following a diagnosis of fetal congenital heart disease; however, there is a very limited evidence base examining how parents actually experience antenatal counselling and make decisions in this context.

**Methods:**

Semi‐structured interviews were conducted with women who had received an antenatal diagnosis of HLHS. Interviews were digitally recorded, anonymised and transcribed verbatim. A thematic content analysis was performed using a constant comparative approach.

**Results:**

Eight mothers of surviving children with HLHS were interviewed. Eight key themes emerged including new perspectives on how women receive antenatal counselling and how it affects their decision making. Three themes in particular are new to the literature: (a) Mothers of children with HLHS reported feelings of intense guilt that arose in the antenatal period around potentially causing the condition in their child. (b) For this group of women, recollections of perceived pessimism during antenatal counselling had a lasting impact. (c) Despite support from partners or extended family, women nevertheless experienced a strong sense that antenatal decision making was largely a ‘maternal’ responsibility.

**Conclusions:**

When recounting their experiences of antenatal counselling, mothers of surviving children with HLHS offer new perspectives that can guide fetal cardiologists in how best to support their individual patients. Further research is needed to comprehensively understand the experience of prospective parents counselled for severe forms of fetal congenital heart disease.

## 1 INTRODUCTION

An antenatal diagnosis of a severe congenital anomaly such as hypoplastic left heart syndrome (HLHS) often requires parents to assimilate a large amount of complex information at a time of intense emotional and psychological distress. However, there is little empirical evidence available from parents who have faced these experiences. A better understanding of the challenges faced by parents in these circumstances may help to inform a more relevant, supportive and intuitive model for antenatal counselling.

Hypoplastic left heart syndrome is a rare form of congenital heart disease in which the left side of the heart fails to develop normally in utero. As a result, there is obstruction to blood flow through the left side of the heart. The syndrome varies in its presentation and encompasses underdevelopment of the mitral valve, left ventricle, aorta and aortic arch. HLHS has been reported to occur in approximately 0.016% to 0.036% of all live births.^1^ There are several options available in the management of this condition: a three‐staged reconstructive surgical technique, termination of the pregnancy and compassionate supportive therapy only. All surgical options are non‐curative or ‘palliative’ procedures, with the ultimate goal of producing a ‘single ventricle’ or ‘Fontan’ circulation, whereby the right ventricle ejects to the aorta and pulmonary blood flow is driven by central venous pressure alone. This strategy carries significant peri‐operative and interstage mortality risks and is associated with long‐term morbidity, including exercise intolerance, ventricular dysfunction, arrhythmias, protein‐losing enteropathy, plastic bronchitis and neurodevelopmental impairment.^3^, ^4^ Stage one of this approach, generally termed the ‘*Norwood Procedure’*, involves enlargement of the atrial septal defect, connection of the aorta to the pulmonary artery, aortic arch reconstruction and the insertion of a systemic‐to‐pulmonary shunt.^2^ Despite some recent debate about the role of compassionate care in HLHS,^5^ the consensus remains that this is a potentially life‐limiting condition with no cure and that treatment decisions should be guided wherever possible by the wishes of the family.

Several studies^6^, ^7^, ^8^ have been conducted looking at the parental experience of caring for a child with HLHS but little work exists on the experience of diagnosis and prenatal counselling. Previous studies have looked at parental decision making in the neonatal period^9^ and yet increasingly, due to improvements in prenatal screening, the diagnosis of HLHS is made antenatally.^10^ This is potentially a critical moment for a pregnant couple, and one which may have a significant impact on how they will continue to interact with health‐care professionals thereafter. It has previously been emphasised that the information given at diagnosis, the manner in which it is presented, and the parents' understanding and interpretation of that information are critical factors in shaping parents' perceptions and management decisions.^11^ Antenatal counselling in HLHS therefore represents a unique window in which doctors have a responsibility to communicate effectively and sensitively with parents in order to support their decision making.

The 2014 review of Children's Heart Surgery Services in Leeds^12^ sought feedback from families and highlighted antenatal diagnosis and care as a key area for improvement. Families felt that the counselling and support provided following the diagnosis of the congenital heart condition was inadequate, and there was a perception of little compassion or understanding. Similarly in the United States, parents receiving a diagnosis of congenital heart disease report sometimes perceiving their doctors to be lacking in compassion and empathy.^11^, ^13^, ^14^ Parents' perception of the information communicated to them about an antenatal diagnosis, as well as the manner in which that information is communicated to them by health‐care professionals, has been shown to impact on their ability to cope with the diagnosis.^15^ Similarly, how parents conceptualise the decision‐making process, and their role within it, will also impact on how they cope in the longer term.^16^

In this study, we sought to interview parents with an antenatal diagnosis of HLHS, using their first‐hand accounts of the decision‐making process to explore the impact of prenatal counselling on how these difficult decisions are navigated in the antenatal period.

## 2 DESIGN & SETTING

This was a single‐centre interview study with parents. Potential participants (parents of patients with a diagnosis of HLHS) were identified using the fetal cardiology database and eligible parents who received a diagnosis of HLHS during their pregnancy from 1995 to 2014 were invited to participate. A purposive sampling strategy was used. We chose this timeframe owing to the fact that 1995 was the year that Guy's and St. Thomas' Hospital started offering the Norwood procedure, as part of a three‐staged surgical intervention for HLHS.

Both parents who had a surviving child with HLHS and mothers who had opted for a termination of pregnancy were approached. Participants were invited to participate by letter and provided with detailed patient information sheets about the study and a copy of the interview topic guide. Written consent was obtained prior to the interviews. Semi‐structured interviews were digitally recorded, anonymised and transcribed verbatim. Topics covered included parental understanding of HLHS, parental memories of being given the diagnosis and discussions that took place at the time, factors which influenced the parental decision‐making process, the doctor's role in the decision‐making process and the ongoing impact of the diagnosis on the parents' and child's life.

Interviews lasted on average 50 mins, ranging between 26 minutes and 1 hour 26 minutes. All interviews were undertaken by SB.

Data were analysed using a thematic content analysis employing a constant comparative approach. Open coding of early data was performed to generate categories. This enabled the range of concepts used by participants to be identified and to extend the analysis so the research question could better be understood in terms of ideas from the data itself. An inductive approach was thus applied such that coding of extracts and a constant comparative approach enabled the team to develop concepts and connections and bring these together into the themes presented. A reflective diary was completed immediately after each interview to provide additional context. Three randomly selected interviews were coded separately by MB‐L and compared across the team. Consensus on emergent themes was reached through regular discussions. Using an iterative process and in‐depth conversation amongst the coders and co‐authors, it was determined that saturation was achieved well before the study ended. Illustrative quotes are presented in the findings.

Ethical approval was granted by the NRES Committee South East Coast—Surrey (REC Reference 14/LO/1557).

### 2.1 Participants

Given concerns from the clinical team that some families would find contact from the research team upsetting, the clinicians were invited to compile a list of patients they felt were eligible and able to participate. Families who the clinical team felt were too vulnerable to be approached were excluded from the list of potential participants. A total of nineteen families were invited to participate in the study. Eight mothers were interviewed between December 2014 and November 2015, all of who had a surviving child with HLHS. Six families who had chosen a termination of pregnancy were invited to participate but we received no responses from this group.

The women had an average age of 31 years (range 20‐41 years) at the time of interview and an average age of 26 years (18‐36 years) at the age of diagnosis. Two of the mothers were separated from the child's father at the time of interview. Parents came from a broad spectrum of educational backgrounds and four of the eight women had a history of working in a health‐care setting, including care homes; none had ever worked in patient‐facing primary or secondary care. None of the families had other children with a history of congenital heart disease. For four of the eight families, the affected child was their first liveborn baby; the other four had between 1 and 2 previous children. Three of the eight families had gone on to have subsequent children after their child with HLHS. Demographic details for participants are summarised in Table 1.

**TABLE 1:** Demographic details of participants

Total number of participants (n)	8
Mean age at interview (range)	31 (20‐41)
Mean age at diagnosis (range)	26 (18‐36)
Maternal Education (n)	
Higher Education and professional/vocational equivalents	2
A levels, vocational level 3 and equivalents	2
GCSE/O Level grade A*‐C, vocational level 2 and equivalents	3
No qualifications	1
Maternal Occupation (n)	
Employed full time	2
Employed part time	1
Homemaker	5
Number of children at the time of diagnosis (n)	
None	4
One	3
Two	1

## 3 RESULTS

Thematic analysis of the interview data revealed eight key themes as detailed below.

### 3.1 Emotional distress and feelings of guilt

Mothers talked about the profound emotional distress they experienced at the time of diagnosis and this was often accompanied by feelings of guilt. Mothers expressed worry about their potential role in causing the fetal heart condition. The mothers saw their own personal health and behaviours as being inextricably linked to the health of their fetus. For many women, these feelings persisted long after their child was born, despite reassurances to the contrary.

…it was so horrific, and I think I kind of beat myself up a lot over it because I thought, oh my gosh, and again blame myself because being diabetic I have that increased chance of having cardiac children and (…) so I'm well aware it is my fault. (Participant 2)

…it was scary and terrifying, and I felt guilty, I felt “why's this happening to me?”, (…), I don't smoke, I didn't drink, I didn't do drugs, I sort of took it for granted that I was going to have a healthy baby, I was young, I'd done everything right so it was just such a shock. (Participant 4)

### 3.2 Determination to understand the condition

Having been given the diagnosis of HLHS mothers discussed how overwhelming they found the information given to them and how difficult it was to understand all of the details about the condition and the treatment options presented to them. This sensation of being overwhelmed was met with a sense of determination to better understand the condition in order to better inform their decision making. Mothers responded by seeking out information: from leaflets, from the internet or from other families affected by HLHS. They frequently emphasised the importance of other people's stories in helping them come to an understanding of their own situation.

…so it was just so, it's all one big blur. It's just so much to take in and like understand. (Participant 7)

(...)that week I just was closed down, I just wanted to search and make sure I was doing the right thing. (...)I just wanted to be like on my own or just constantly on the internet, just looking at different stories and different views. (Participant 1)

### 3.3 Value of clear explanations

Many mothers spoke of the positive experience they felt they had at the tertiary referral centre and much of this was attributed to feeling that health professionals gave clear and detailed explanations. They valued clinicians who took time to explain things repeatedly if needed, to respond to questions and to clarify anything they were unsure of.

(...)they went through it over and over again, they did diagrams, they gave us information for charities (…) I wouldn't have changed a thing because it helped us so much. If they hadn't have done all that I don't think I would have coped (Participant 3)

(...)he sort of sat down and talked it all through with us and did draw diagrams but with him, I felt like he had more time. I felt like basically he would sit there for as long as we needed him to sit there. And answer whatever questions we needed and repeat it as many times as I needed him to until it sort of went into my head a bit more. And that was really good.(Participant 8)

### 3.4 Recollections of perceived pessimism

A number of the women spoke about feeling offended by the way the diagnosis was presented to them for the first time at their local hospital. Often this related to the prognosis being presented in an overly negative way or a perception that the treatment options were not presented in the appropriate order. When initial counselling was offered by the local obstetric team prior to referral to the tertiary fetal cardiology centre, women frequently perceived health professionals to be pessimistic towards the diagnosis of HLHS. This early counselling clearly had a deleterious impact even when women spoke of feeling more positive after consultation in the tertiary centre.

(...)but I felt at that time that everything was just negative, it was very negative and I understand that you have to be told the worst case scenario but to me it was just all negative (Participant 6)

(...)she was very sensitive in how she was talking about it but, um, the options that she gave me were given the wrong way. I believe that they were given the wrong way because the first option, she, the first thing she offered me was an abortion (…) it shouldn't be the first option. (Participant 5)

### 3.5 A sense of responsibility for decision making

The mothers interviewed expressed how the weight of a decision to pursue a termination of pregnancy would have been difficult for them to bear. It was clear, however, that they felt a sense of responsibility as parents, to be the ones making the decision for their unborn child, and that this was not a decision they saw as appropriate for health professionals or other families to make on their behalf. Many mothers spoke of how much they valued not being pressurised by the doctors who counselled them and feeling that the decision was entirely up to them as parents.

Yeah, it was my decision, it wasn't their decision and it was never their, like they never made me feel like they was saying “she needs it now and we're making the decision over you” so it was always left to me to decide what I wanted to do rather than being pushed into anything.(Participant 5)

### 3.6 Maternal responsibility

Whilst mothers reported discussing the decision to continue with the pregnancy with their partner and others, their sense of nevertheless needing to make the decision alone was surprisingly strong. The choice to pursue a medical termination is ultimately, from a legal standpoint, that of the mother alone. The mothers interviewed reflected this profound sense of a ‘maternal’ responsibility in the decision‐making process, as opposed to a shared ‘parental’ responsibility. This was often tied to the realisation that they would be acting as the principal carer for the child after birth.

I mean ultimately it's my body I can do what I want. But obviously as parents it's me and (the father). (Participant 2)

Because I knew that it was going to be me, being the main, being the carer, because obviously my husband is out at work. (Participant 8)

### 3.7 Giving the child a chance

Seven of the eight mothers interviewed spontaneously spoke about the concept of ‘giving their child a chance’. However difficult the odds, these mothers felt compelled to keep all options open to their offspring.

My son deserved a chance even if the chances weren't very good, yeah, he just needed that. (Participant 2)

I had to give her that chance because she could well be my one and only so yeah, I just went from there really. (Participant 5)

### 3.8 Family ethics

Some of the mothers touched on their feelings of responsibility to the wider family and the realisation that continuing with the pregnancy with a child affected by HLHS would have implications for their existing children. Nonetheless, for this group, their responsibility to existing children was conceptualised at an equal level to their responsibility to the fetus and often brought together into a view of the needs of the family as a whole.

…obviously what was important as well, I had to think about my older son as well… because I had to think what he would be like with all of this. It hasn't been easy on him (Participant 1)

## 4 DISCUSSION

This study presents perspectives from eight women with surviving children with a prenatal diagnosis of HLHS. The age of their children at the time of interview ranged from 1 to 11 years of age. Despite the multiple high‐risk surgeries and medical treatments for their child in the intervening period, each retained vivid recollections of the emotional and psychological impact of their antenatal diagnosis and the experience of prenatal counselling.

The role of the clinician, in this case the fetal cardiologist, is to impart a significant amount of important information to the parents in order to best prepare them to make the right decision for their family as to how to progress with the pregnancy. In the case of a severe congenital heart disease such as HLHS, this includes preparing them for the reality of a lifetime of medical care, multiple surgeries and the possibilities of disability or even death. Yet they are also forging a pivotal relationship with that family, and the quality of that relationship and the memories of these early consultations will have a lasting impact.

The emotional distress at receiving a severe antenatal diagnosis such as HLHS cannot be overestimated and the mothers in this study spoke openly about their shock, fear and sadness at the time of diagnosis. However, what is particularly poignant in these narratives is the feelings of intense guilt shouldered by mothers, who often blame themselves for causing the condition. This emphasises a crucial role for clinicians when counselling, to seek to reassure and assuage any feelings of guilt at an early stage. The women in this study described their impulse to thoroughly understand and research the diagnosis. This echoes previous work which found *‘information seeking’* to be a prominent mechanism used by some parents to overcome uncertainty when faced with a prenatal diagnosis of a severe congenital anomaly.^17^

The strong recollections of perceived pessimism during early antenatal counselling is another important finding. Whilst it is always the clinicians' responsibility to offer all treatment options available to the patient, the manner and even the order in which these options are presented may have an important influence on later perceptions of counselling. In particular, initial counselling at screening (ie that undertaken before tertiary cardiology review) should be extremely circumspect with regard to the diagnosis, prognosis and management options.

The mothers in this group felt a strong sense of being personally responsible for decision making and valued the fact that their clinicians did not make them feel pressured into any particular treatment path. When faced with making decisions about their pregnancy, the women in this group favoured neutrality and a non‐directive approach from their clinician. This further supports previous findings that whilst some health‐care professionals may perceive these decisions as being too burdensome for parents to make, parents themselves often conceptualise such decisions as part of their parental responsibility, and may feel offended when professionals provide information in what the parents perceive as a biased manner.^18^, ^19^

Interestingly the women in our group emphasised how beyond them identifying a ‘parental’ responsibility for decision making, they felt a strong sense of it being a ‘maternal’ decision. This was linked to their sense of ownership over treatment decisions about their own body but also to their anticipated role as main carers for their future child. We do not know how generalisable this finding is across all women.

In the cohort interviewed, all with continuing pregnancies, the reasons given for this decision were largely unanimous in their formulation of the desire ‘to give their child a chance’. Here, we see that the mothers in this group felt an early sense of responsibility to advocate for their future child by keeping all options open. Research has shown that parents of children with life‐threatening illnesses often feel driven by a need to leave no stone unturned, and hence, they will pursue treatment options relentlessly, even those that may have low odds of success.^20^ Another study that considered how parents make the choice to pursue surgery for HLHS also found that a sense of hope and seeking a way to ‘fix’ the problem were key motivators for the decision.^21^ How parents choose to advocate for their future child is an interesting area for further research, and undoubtedly one that cannot be answered in full without the perspective of those who chose a termination or compassionate care.

The women in our group spoke about how considerations about existing family members came into their process of decision making. This is a crucial insight into some of the values that influence how parents make decisions in the antenatal period. It has been argued that the historical approach to antenatal counselling that focuses on the provision of precise epidemiologic outcome data is incomplete as it neglects the inherent variability in the values that shape a parent's interpretation of that data. Understanding the values that influence parental decision making and developing the communication skills to assist parents in articulating their values is arguably the best strategy, we have to improve our support for families.^22^

Finally, this work reinforces the notion that the relationship between parents and clinicians is critical to decision making.^23^ With this in mind, clinicians may be able to better support the antenatal decision‐making process by considering the nature of this relationship before, during and after counselling; seeking to understand at an early stage of the process how parents may view their potential options. By making space for a pregnant couple to lay out their views first, the clinician may be better able to assess their practical, psychological and emotional needs, and tailor their counselling accordingly.

### 4.1 Study limitations

Whilst both parents were invited to participate in the study, ultimately our interviews were solely with mothers, leaving the paternal voice out of the picture. Very limited research exists as to the father's experience of an antenatal diagnosis of a severe congenital anomaly. Existing evidence suggests that fathers set their own needs aside to attend to the supportive needs of their pregnant partner whilst focusing on coming to a joint decision regarding whether to continue or terminate the pregnancy.^24^

We were only able to recruit families who had chosen to continue with the pregnancy following the diagnosis of HLHS and all had a surviving child at the time of interview. This data therefore only represents a very specific cohort of parents and can only offer perspectives on how some women experience antenatal counselling and decision making. Notably absent from this study are women who opted for termination of pregnancy, representing a significant limitation to some of the findings in this work. 8 out of the 13 families with surviving children with HLHS elected to participate versus 0 out of the 6 families who had chosen a termination of pregnancy. Further research is needed into how parents who choose termination or compassionate supportive care following a diagnosis of HLHS experience the decision‐making process.

Interviews were conducted retrospectively with a mean time from the child's birth of 65 months. The mothers' account of their antenatal decision‐making process is hence subject to recall bias and their narrative will undoubtedly have been shaped by their subsequent experience of caring for a child with HLHS.

Furthermore, the field of fetal medicine and treatment of congenital heart disease is a rapidly changing area and we cannot assume that for any pregnant women there will be unity in their views or experiences. Nonetheless, there is value in trying to elucidate the underpinnings of how these women made decisions during their pregnancy.

### 4.2 Future work

Despite considerable advances in antenatal diagnostic technologies in recent years, there is a paucity of research examining parental experiences in‐depth.^25^ This study highlights that by better understanding the experience of prospective parents undergoing antenatal counselling and by seeking to comprehend how they make difficult decisions during this time, we can better understand what is helpful and what is unhelpful coming from health‐care professionals. By understanding the decision‐making process for families, we can better enrich the relationship between parents and clinicians and work towards more tailored and intuitive counselling. These questions would be best answered by prospective research into antenatal decision making which could capture the experience of all parents faced with a similar diagnosis.

## 5 CONCLUSIONS

When recounting their experiences of antenatal diagnosis of a severe congenital heart defect, mothers of surviving children with HLHS offer unique insights into the process of antenatal decision making. Training and guidance for those undertaking prenatal counselling in congenital heart disease should take into account these findings. There is a need for further prospective work into how parents with an antenatal diagnosis of severe congenital heart disease navigate treatment decisions with their clinicians in order to improve and individualise antenatal counselling.

## CONFLICT OF INTEREST

We declare no competing interests.

## ETHICAL APPROVAL

Ethical approval was granted by the NRES Committee South East Coast—Surrey (REC Reference 14/LO/1557).

## Data Availability

The data that support the findings of this study are available on request from the corresponding author. The data are not publicly available due to privacy or ethical restrictions.
